# Association between *CHEK2* H371Y mutation and response to neoadjuvant chemotherapy in women with breast cancer

**DOI:** 10.1186/s12885-015-1203-3

**Published:** 2015-03-28

**Authors:** Yin Liu, Ye Xu, Tao Ouyang, Jinfeng Li, Tianfeng Wang, Zhaoqing Fan, Tie Fan, Benyao Lin, Yuntao Xie

**Affiliations:** Key Laboratory of Carcinogenesis and Translational Research (Ministry of Education), Breast Center, Beijing Cancer Hospital & Institute, Peking University Cancer Hospital, Beijing, 100142 People's Republic of China

## Abstract

**Background:**

Our previous study suggested that the recurrent *CHEK2* H371Y mutation is a novel pathogenic mutation that confers an increased risk of breast cancer. The purpose of this study was to investigate whether breast cancer patients with *CHEK2* H371Y mutation were more likely to respond to neoadjuvant chemotherapy.

**Methods:**

We screened a cohort of 2334 Chinese women with operable primary breast cancer who received a neoadjuvant chemotherapy regimen for *CHEK2* H371Y germline mutations. Pathologic complete response (pCR) was defined as the absence of tumor cells in the breast after the completion of neoadjuvant chemotherapy.

**Results:**

Thirty-nine patients (1.7%) with *CHEK2* H371Y germline mutation were identified in this cohort of 2334 patients. *CHEK2* H371Y mutation carriers had a significantly higher pCR rate than non-carriers (33.3% versus 19.5%, *P* = 0.031) in the entire study population, and *CHEK2* H371Y mutation-positive status remained an independent favorable predictor of pCR in a multivariate analysis (odds ratio [OR] = 3.01; 95% confidence interval [CI]: 1.34- 6.78, *P =* 0.008). *CHEK2* H371Y carriers had a slightly worse distant recurrence-free survival than non-carriers (adjusted hazard ratio [HR] =1.24, 95% CI: 0.59-2.63).

**Conclusions:**

*CHEK2* H371Y mutation carriers are more likely to respond to neoadjuvant chemotherapy than are non-carriers.

## Background

*CHEK2* (Cell-cycle-checkpoint kinase 2, also known as *CHK2*) encodes a multifunctional kinase that is activated mainly by the ataxia-telangiectasia mutated (ATM) protein in response to DNA double-strand breaks [1-4]. Activated *CHEK2* in turn phosphorylates several critical cell-cycle proteins, including p53, Cdc25 and BRCA1, which trigger cell-cycle arrest, apoptosis, and the activation of DNA repair [5-7].

Numerous studies have demonstrated that *CHEK2* is a moderate breast cancer susceptibility gene [8-12]. *CHEK2* 1100delC, a truncating mutation that abrogates the kinase activity of the protein, confers an approximately 2-fold increase in breast cancer risk [8,13-15]. However, the prevalence of *CHEK2* 1100delC mutation varies widely among ethnic groups [8,11,16-19]. The mutation is mostly found in the Dutch population [8,20], and it is absent or very rare in other populations [16-19]. We previously screened 2255 Chinese women (1027 breast cancer cases and 1228 healthy controls) for *CHEK2* 1100delC and failed to find this mutation in this population. However, a novel recurrent *CHEK2* mutation near the *CHEK2* 1100delC mutation, *CHEK2* 1111C > T (H371Y), was found in Chinese women [21]. *CHEK2* H371Y is within the activation loop of the *CHEK2* protein kinase domain, which is essential for the activation of *CHEK2* in response to DNA damage. Functional analysis reveals that the *CHEK2* H371Y mutation produces a dramatic decline in *CHEK2* activity and is a pathogenic mutation [21]. *CHEK2* H371Y confers a 2.43-fold increase in breast cancer risk in Chinese women.

The disruption of *CHEK2* kinase activity may not only contribute to breast cancer development but also influence breast cancer survival or response to the adjuvant therapy. Two studies have suggested that the *CHEK2* 1100delC mutation is associated with poor recurrence-free survival in breast cancer [22,23], indicating that patients with *CHEK2* 1100delC mutation have an aggressive phenotype.

No previous studies have investigated the association between *CHEK2* germline mutation and response to neoadjuvant chemotherapy in breast cancer. Therefore, in the current study, we investigated whether *CHEK2* H371Y mutation carriers are more likely to respond to neoadjuvant chemotherapy in terms of pathologic complete response (pCR) in a large cohort of 2334 breast cancer patients who received neoadjuvant chemotherapy and further explored the association between *CHEK2* H371Y mutation status and distant recurrence-free survival (DRFS).

## Methods

### Study population

A total of 2382 operable primary breast cancer patients with stage I-III were treated with neoadjuvant chemotherapy at the Breast Center of Peking University Cancer Hospital from October 2003 to December 2010. The mean age of the subjects was 47.6 years (range, 22–75 years). Tumor stage was classified according to the tumor-node-metastasis classification of the Union Internationale Contre le Cancer. Tumor size was defined as the maximum tumor diameter measured on the mammogram and/or ultrasonogram at the time of diagnosis. The tumors were graded according to the modified Bloom-Richardson system. Written consent was obtained from all subjects. This study was approved by the Research and Ethical Committee of Peking University Cancer Hospital.

### *CHEK2* H371Y germline mutations

Peripheral blood samples were collected from all patients. Genomic DNA was extracted from the leukocyte pellet by proteinase K digestion followed by phenol-chloroform extraction. The *CHEK2* H371Y mutation was detected by polymerase chain reaction (PCR) followed by denaturing high-performance liquid chromatography (DHPLC) and sequencing or directed sequencing as described previously [21]. We screened all 2382 patients for the germline *CHEK2* H371Y mutation. *CHEK2* H371Y status was not readable for 46 patients, and 41 of the 2336 patients were found to carry the mutation. We then screened these 41 *CHEK2* H371Y mutation carriers for germline mutations in *BRCA1/2*; two patients also carried a *BRCA2* germline mutation and were excluded from this study. Therefore, 2334 patients, 39 of whom were *CHEK2* H371Y carriers, were included in the final analysis in the current study.

### Estrogen receptor (ER), progesterone receptor (PR), and HER2 status

ER, PR, and HER2 status were determined in the core-needle biopsy breast cancer tissue obtained before the initiation of neoadjuvant chemotherapy as described previously [24].

### Neoadjuvant chemotherapy regimens

Among the 2334 patients who received neoadjuvant chemotherapy, 94% received 4–8 cycles. Treatments were categorized in three subgroups as follows:

1. 859 patients received an anthracycline-based regimen. The detail of the regimens are described previously [24]. Of these, 537 patients received a CTF regimen; 247 patients received an FEC regimen; 59 patients received a CAF regimen; the remaining 16 patients received other types of anthracycline regimens.
2. 882 patients received an anthracycline-taxane containing regimen. Of these, 682 patients received two cycles of anthracycline followed by 4 cycles of paclitaxel alone (80 mg/m^2^ IV once per week for 12 weeks) or paclitaxel plus carboplatin (paclitaxel 175 mg/m^2^ IV on day 1 or paclitaxel 60 mg/m^2^ IV on day 1, day 8, and day 15, and carboplatin AUC 6 IV on day 1 every three weeks); 181 patients received 4 cycles of paclitaxel alone or docetaxel plus cyclophosphamide (docetaxel 75 mg/m^2^ IV on day 1 and cyclophosphamide 600 mg/m^2^ IV on day 1 every three weeks), followed by 2 to 4 cycles of anthracyclines. The remaining 19 patients received other types of anthracycline/taxane containing regimens, i.e., a TE regimen (docetaxel plus epirubicin) or TAC regimen (docetaxel, doxorubicin, and cyclophosphamide).
3. 593 patients received a taxane-based regimen without anthracyclines. Of these, 494 patients received 4 cycles of paclitaxel (80 mg/m^2^ IV once a week for 12 weeks); 76 patients received paclitaxel plus carboplatin (paclitaxel 60 mg/m^2^ IV on day 1, day 8, and day 15, and carboplatin AUC 6 IV on day 1 every three weeks). The remaining 23 patients received docetaxel plus cyclophosphamide (docetaxel 75 mg/m^2^ IV on day 1 and cyclophosphamide 600 mg/m^2^ IV on day 1 every three weeks).

In this cohort of 2334 patients, 108 patients received intravenous trastuzumab in combination with neoadjuvant chemotherapy.

After completion of neoadjuvant chemotherapy, patients were treated with mastectomy (n = 1351) or breast-conserving surgery (n = 983) depending on the tumor size, presence of multiple lesions or patient preference. pCR was defined as the absence of invasive breast cancer cells in the breast after the completion of neoadjuvant chemotherapy [25,26].

Sixty-two percent of patients received adjuvant chemotherapy with the same or alternative regimens after operation; patients with axially positive lymph nodes and/or breast-conserving therapy received radiotherapy; patients with ER and/or PR-positive disease received endocrine therapy (20 mg/d tamoxifen for 5 years or 1 mg/d anastrozole for 5 years).

### Statistical analysis

The differences in clinicopathological characteristics between *CHEK2* H371Y carriers and non-carriers were determined by Pearson’s chi-squared test. The associations between *CHEK2* H371Y mutation status, clinicopathologic characteristics, and pathological response to neoadjuvant chemotherapy were determined by Pearson’s chi-squared test or Fisher’s exact test when the number of patients was small. A logistic regression model was applied to determine whether a factor was an independent predictor of pCR in a multivariate analysis. Distant recurrence-free survival (DRFS) was defined as the time from the date of diagnosis to first distant recurrence (not including second primary malignancies) or death from breast cancer without a recorded relapse. Survival curves were derived from Kaplan–Meier estimates and compared using log-rank tests. All statistical tests were two-sided, and P values <0.05 were considered statistically significant. The statistical analyses were performed using SPSS 16.0 software (Chicago, IL, USA).

## Results

### Patient and tumor characteristics

In this cohort of 2334 patients, 39 women carried a *CHEK2* H371Y mutation (39/2334, 1.7%). Since the primers used in this study covered the *CHEK2* 1100delC mutation, none *CHEK2* 1100delC was found in the current study. The clinicopathological characteristics and chemotherapy regimens of each group are presented in Table 1. No significant differences were found between *CHEK2* H371Y carriers and non-carriers with regard to tumor size, lymph node status, ER or PR status, chemotherapy regimens, surgery type, tumor grade, and pathological type (Table 1). However, *CHEK2* carriers were more likely to be diagnosed at or before age of 50 as compared with non-carriers (*P =* 0.036, Table 1), and *CHEK2* carriers were less likely to be HER2-positive (15.4%) than non-carriers (30.8%) (*P =* 0.038, Table 1).

**Table 1:** **Association of patient/tumor characteristics with** ***CHEK2*** **H371Y mutation status**

Characteristics	n	Carriers, n (%)	Non-carriers, n (%)	*P-*value
Total	2334	39 (1.7)	2295 (98.3)	
Age				0.036
≤50y	1415	30 (76.9)	1385 (60.3)	
>50y	919	9 (23.1)	910 (39.7)	
ER				0.30
Positive	1365	26 (66.7)	1339 (58.3)	
Negative	969	13 (33.3)	956 (41.7)	
PgR				0.26
Positive	1108	22 (56.4)	1086 (47.3)	
Negative	1226	17 (43.6)	1209 (52.7)	
HER2				0.038
Positive	714	6 (15.4)	708 (30.8)	
Negative	1620	33 (84.6)	1587 (69.2)	
TNBC				0.61
Non-TNBC	1837	32 (82.1)	1805 (78.6)	
TNBC	497	7 (17.9)	490 (21.4)	
Tumor size				0.70
<2 cm	786	12 (30.8)	774(33.7)	
≥2 cm	1548	27 (69.2)	1521(66.3)	
Lymph node				1.00
Positive	1014	17 (43.6)	997 (43.6)	
Negative	1314	22 (56.4)	1292 (56.4)	
Unknown	6		6	
Nuclear grade				
1	199	6(17.6)	193 (9.7)	0.29
2	1518	24(70.6)	1494 (75.3)	
3	302	4(11.8)	298 (15.0)	
Unknown	315	5	310	
Histology				0.45
Ductal	2064	33 (84.6)	2031 (88.5)	
Others	270	6 (15.4)	264 (11.5)	
Chemotherapy type				0.27
A-based,without a T	859	18 (46.2)	841 (36.6)	
A-T containing	882	10 (25.6)	872 (38.0)	
T-based,without a A	593	11(28.2)	582(25.4)	
Trastuzumab use				0.42
Yes	108	3 (7.7)	105(4.6)	
No	2226	36 (92.3)	2190(95.4)	
Surgery type				
BCS	983	22 (56.4)	961 (41.9)	0.07
Mastectomy	1351	17 (43.6)	1334 (58.1)	

ER, Estrogen receptor; PgR, Progesterone receptor; HER2, Human epidermal growth factor receptor-2; A, Anthracycline; T, Taxane; BCS, Breast-conserving surgery.

### Response to neoadjuvant chemotherapy in CHEK2 carriers and non-carriers

Overall, 460 patients (19.7%) achieved a pCR after neoadjuvant chemotherapy. The pCR rate was 33.3% (13/39) for *CHEK2* H371Y mutation carriers and 19.5% (447/2295) for non-carriers (Table 2), indicating that *CHEK2* carriers had a significantly higher pCR rate than did non-carriers (*P =* 0.031; Table 2). In a univariate analysis, other factors associated with improved pCR rates were ER negativity (*P <* 0.001), PR negativity (*P <* 0.001), HER2 positivity (*P <* 0.001), tumor size ≤2 cm (*P <* 0.001), negative lymph nodes (*P <* 0.001), and high tumor grade (*P <* 0.001). The pCR rate was significantly higher in patients who received trastuzumab (43.5%) in combination with neoadjuvant chemotherapy compared with patients who did not (18.6%; *P <* 0.001) (Table 2).

**Table 2:** **pCR Rates by clinical characteristics**

Characteristic	N	non-pCR, n (%)	pCR, n (%)	*P-*value
Total	2334	1874 (80.3)	460 (19.7)	
Age				0.49
≤50y	1415	1129 (79.8)	286 (20.2)	
>50y	919	745 (81.1)	174 (18.9)	
ER				<0.001
Positive	1365	1202 (88.1)	163 (11.9)	
Negative	969	672 (69.3)	297 (30.7)	
PgR				<0.001
Positive	1108	984 (88.8)	124 (11.2)	
Negative	1226	890 (72.6)	336 (27.4)	
HER2				<0.001
Positive	714	521 (73.0)	193 (27.0)	
Negative	1620	1353 (83.5)	267 (16.5)	
TNBC				<0.001
Non-TNBC	1837	1517 (82.6)	320 (17.4)	
TNBC	497	357 (71.8)	140 (28.2)	
Tumor size				
<2 cm	786	596 (75.8)	190 (24.2)	<0.001
≥2 cm	1548	1278 (82.6)	270 (17.4)	
Lymph node				
Positive	1014	892(88.0)	122 (12.0)	<0.001
Negative	1314	978(74.4)	336 (25.6)	
Nuclear grade				
1	199	174 (87.4)	25 (12.6)	<0.001
2	1518	1252 (82.5)	266 (17.5)	
3	302	184 (60.9)	118 (39.1)	
Histology				0.60
Ductal	2064	1654 (80.1)	410 (19.9)	
Others	270	220 (81.5)	50 (18.5)	
*CHEK2* H371Y				0.031
Non-carriers	2295	1848 (80.5)	447 (19.5)	
Carriers	39	26(66.7)	13 (33.3)	
Chemotherapy type				0.50
A-based,without a T	859	700 (81.5)	159 (18.5)	
A-T containing	882	705 (79.9)	177 (20.1)	
T-based,without a A	593	469 (79.1)	124 (20.9)	
Trastuzumab use				<0.001
Yes	108	61 (57.1)	47 (43.5)	
No	2225	1812 (81.4)	413 (18.6)	
Surgery type				<0.001
BCS	983	756 (76.9)	227 (23.1)	
Mastectomy	1351	1118 (82.8)	233 (17.2)	

pCR, pathologic complete response; ER, Estrogen receptor; PgR, Progesterone receptor; HER2, Human epidermal growth factor receptor-2; A, Anthracycline; T, Taxane; BCS, Breast-conserving surgery.

In the multivariate logistic regression model, *CHEK2* H371Y mutation-positive status (odds ratio [OR] = 3.01; 95% confidence interval [CI]:, 1.34 to 6.78; *P* =0.008), high tumor grade (OR = 2.28; 95% CI: 1.71 to 3.03; *P <* 0.001), tumor size less than 2 cm (OR = 1.76; 95% CI: 1.38 to 2.24; *P =* <0.001), negative lymph nodes (OR = 2.10; 95% CI: 1.63 to 2.71; *P <* 0.001), ER-negativity (OR = 1.99; 95% CI, 1.48 to 2.67; *P <* 0.001), PR-negativity (OR = 1.65; 95% CI: 1.21 to 2.26; *P =* 0.002), and concurrent trastuzumab use (OR = 2.42; 95% CI: 1.47 to 3.99; *P =* 0.001) were independent significant predictors of pCR (Table 3).

**Table 3:** **Multivariate logistic regression model for pathological complete response**

Variable	Pathological complete response (pCR)		*P-*value
		OR (95% CI)	
*CHEK2* H371Y	(MT *v* WT)	3.01 (1.34-6.78)	0.008
Age	(≤50 *v* >50 years)	1.26 (0.99-1.61)	0.06
Tumor grade	(III *v* I + II)	2.28 (1.71-3.03)	<0.001
Tumor size	(<2 *v* ≥2 cm)	1.76 (1.38-2.24)	<0.001
Lymph nodes	(Negative *v* Positive)	2.10 (1.63-2.71)	<0.001
ER	(Negative *v* Positive)	1.99 (1.48-2.67)	<0.001
PgR	(Negative *v* Positive)	1.65 (1.21-2.26)	0.002
HER2	(Positive *v* Negative)	1.36 (1.05-1.75)	0.021
Trastuzumab use	(Yes *v* No)	2.42 (1.47-3.99)	0.001

ER, Estrogen receptor; PgR, Progesterone receptor; HER2, human epidermal growth factor receptor.

In the anthracycline-treated subgroup, *CHEK2* mutation carriers had a higher pCR rate than non-carriers (27.8% *vs* 18.3%), but this was not statistically significant (*P =* 0.35). In the anthracycline/taxane-treated subgroup, *CHEK2* mutation carriers showed a significantly higher pCR rate than non-carriers (50.0% *vs* 19.7%; *P* = 0.032). In the taxane-treated subgroup, *CHEK2* mutation carriers also had a higher pCR rate than non-carriers (27.3% *vs* 20.8%), but this was not statistically significant (*P* = 0.71) (Table 4).

**Table 4:** **Association of** ***CHEK2*** **H371Y with pathological response according to neoadjuvant treatment regimens**

Chemotherapy	N	non-pCR, n (%)	pCR, n (%)	*P-*value
Anthracycline	859			0.35
Non-carriers	841	687 (81.7)	154 (18.3)	
Carriers	18	13 (72.2)	5 (27.8)	
Anthracycline-taxane	882			0.032
Non-carriers	872	700 (80.3)	172 (19.7)	
Carriers	10	5 (50.0)	5 (50.0)	
Taxane	593			0.71
Non-carriers	582	461 (79.2)	121 (20.8)	
Carriers	11	8 (72.7)	3 (27.3)	

pCR, pathologic complete response.

### Survival estimates

**Figure 1:**
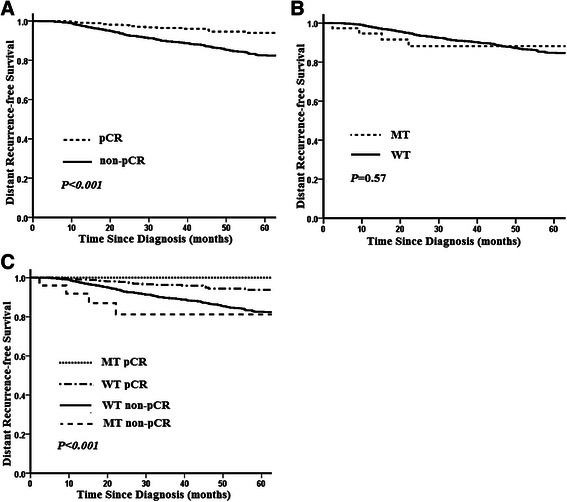
**Kaplan-Meier Estimates of distant recurrence-free survival by pCR and*CHEK2*H371Y mutation status in 2334 breast cancer patients who received neoadjuvant chemotherapy.** Distant recurrence-free survival by pCR status **(A)**; Distant recurrence-free survival by *CHEK2* H371Y mutation status **(B)**; Distant recurrence-free survival by pCR and *CHEK2* H371Y mutation status **(C)**.

Follow-up data were available for all patients, and the median follow-up time was 38 months (range 1 to 104 months). A total of 258 patients (11.1%) experienced a distant metastases or died of breast cancer during the follow-up period. The estimated 5-year distant recurrence-free survival (DRFS) rate for the entire study population was 84.9% (95% CI: 82.9% to 86.9%). Patients who achieved a pCR had a significantly better 5-year DRFS rate than patients who did not (93.5% vs 82.6%, *P <* 0.001) (Figure 1A). *CHEK2* H371Y mutation carriers had a slightly worse DRFS than did non-carriers (adjusted hazard ratio [HR] =1.24, 95% CI: 0.59-2.63), but the difference did not reach significance (*P* = 0.57) (Figure 1B). We then stratified the *CHEK2* carriers and non-carriers by pCR status. The 5-year DRFS rates for *CHEK2* carriers with or without pCR were 100.0% and 81.2%, respectively, whereas the 5-year DRFS rates for non-carriers with or without pCR were 93.6% and 82.7%, respectively (Figure 1C). Patients who achieved a pCR had a better DRFS than who did not in both *CHEK2* mutation carriers and non-carriers (*P <* 0.001). However, *CHEK2* carriers without a pCR exhibited the worst DRFS in the four subgroups (Figure 1C).

## Discussion

In our previous study, we identified a novel recurrent *CHEK2* H371Y mutation in Chinese women, and this mutation decreases *CHEK2* activity and confers an approximately 2.4-fold increase in breast cancer risk [21]. In the present study, we investigated the association between *CHEK2* H371Y and pathologic response in 2334 women who received neoadjuvant chemotherapy. To the best of our knowledge, this is the first study to report that *CHEK2* H371Y mutation carriers are more likely to respond to neoadjuvant chemotherapy than are non-carriers and that the H371Y mutation status was an independent favorable predictor of pCR in a multivariate analysis.

In the subgroup analyses, *CHEK2* H371Y mutation carriers had a higher pCR rate than did non-carriers in both neoadjuvant anthracycline-based regimens and taxane-based regimens, although the difference did not reach statistical significance. However, the *CHEK2* mutation carriers had a significantly higher pCR than did non-carriers among the subgroup of women who received neoadjuvant anthracycline/taxane-containing regimen.

*CHEK2* is involved in various DNA damage responses, including cell-cycle checkpoints, genome maintenance, DNA repair and apoptosis [27]. Anthracyclines induce double-strand DNA breaks [28,29], the repair of which is impaired in the deficiency of *CHEK2* protein [21,30,31]. Tumor cells that express mutated *CHEK2* 347 exhibited a 2-to 4-fold increase in apoptosis upon treatment with adrimycin [6]. *CHEK2* kinase activity is also required for proper mitotic spindle assembly and chromosome stability [32], and *CHEK2* deficient lymphoma cells are more sensitive to taxol [33]. In line with these findings, our results suggested that *CHEK2* H371Y was sensitive to both anthracycline and taxane.

Previous studies showed that breast cancer patients with the *CHEK2* 1100delC mutation had a worse disease-free survival than did patients without this mutation [22,23]. One recent study suggested that breast cancer patients with *CHEK2* 1100delC mutation had a worse survival beyond 6 years after diagnosis than did non-carriers [34]. In the current study, *CHEK2* H371Y carriers showed a slightly poorer DRFS than non-carriers in the entire study cohort with 5-years, it is worth to investigate the survival impact of the *CHEK2* H371Y mutation in a long-term follow-up. However, *CHEK2* carriers or non-carriers who achieved a pCR had a significant better DRFS than those who did not, whereas *CHEK2* H371Y carriers who did not reach a pCR had the worst DRFS in the four subgroups. Although *CHEK2* H371Y carriers were more likely to respond to neoadjuvant chemotherapy, only small subset of mutation carriers achieved a pCR, the majority of *CHEK2* H371Y carriers who did not reach a pCR might have a particularly aggressive phenotype.

Germline *BRCA1* mutation carriers are sensitive to anthracycline or cisplatin neoadjuvant chemotherapy [35-37]; Byrski et al. recently reported that *BRCA1* mutation carriers are extremely sensitive to cisplatin-based neoadjuvant chemotherapy (pCR rate 61%, 65 out of 107 patients) [38]. *CHEK2* H371Y mutation may share some similarity to *BRCA1* mutation, therefore, an interest issue is to see whether *CHEK2* H371Y mutation carriers are responsive to cisplatin. Recent clinical trials showed that *BRCA1* mutation carriers are sensitive to poly (ADP-ribose) polymerase (PARP) inhibitors [39,40]. In vitro studies suggested that tumor cells with silenced *CHEK2* expression showed an increased sensitivity to the PARP 1 inhibitor [33,41]. Therefore, *CHEK2* H371Y mutation carriers may be potential candidates for treatment with PARP1 inhibitors.

Although the entire study population is large, the number of individuals with *CHEK2* H371Y is relatively small; particularly when the mutation carriers were stratified in several treatment groups, therefore, the results are premature and should be interpreted cautiously.

Nevertheless, our study suggests that patients with a deficiency of *CHEK2* activity due to germline mutation, like H371Y, are sensitive to neoadjuvant chemotherapy. It would be of great interest to explore whether other *CHEK2* germline mutations (i.e., *CHEK2* 1100delC) are similarly responsive to neoadjuvant chemotherapy.

## Conclusions

Our results suggest that *CHEK2* H371Y mutation carriers are more likely to respond to neoadjuvant chemotherapy than are non-carriers. In addition, *CHEK2* H371Y mutation may share some similarity to *BRCA1* mutation, therefore, *CHEK2* H371Y mutation carriers may be potential candidates for treatment with PARP1 inhibitors.

## Abbreviations

A: Anthracycline
ATM: Ataxia-telangiectasia mutated
BCS: Breast-conserving surgery
*CHEK2*: Cell-cycle-checkpoint kinase 2
CI: Confidence interval
DHPLC: Denaturing high-performance liquid chromatography
ER: Estrogen receptor
FISH: fluorescence *in situ* hybridization
HER2: Human epidermal growth factor receptor-2
HR: Hazard ratio
IHC: Immunohistochemistry
OR: Odds ratio
PARP: Poly(ADP-ribose) polymerase
pCR: Pathologic complete response
PgR: Progesterone receptor
RFS: Recurrence-free survival
T: Taxane
